# Ta_2_O_5_-TiO_2_ Composite Charge-trapping Dielectric for the Application of the Nonvolatile Memory

**DOI:** 10.1038/s41598-017-05248-6

**Published:** 2017-07-20

**Authors:** C. Y. Wei, B. Shen, P. Ding, P. Han, A. D. Li, Y. D. Xia, B. Xu, J. Yin, Z. G. Liu

**Affiliations:** 1 Department of Materials Science and Engineering, College of Engineering and Applied Sciences, Nanjing University, Nanjing, 210093 China; 2 National Laboratory of Solid State Microstructures and Collaborative Innovation Center of Advanced Microstructures, Nanjing University, Nanjing, 210093 China

## Abstract

The charge-trapping memory devices with a structure Pt/Al_2_O_3_/(Ta_2_O_5_)_*x*_(TiO_2_)_*1*−*x*_/Al_2_O_3_/p-Si (x = 0.9, 0.75, 0.5, 0.25) were fabricated by using rf-sputtering and atomic layer deposition techniques. A special band alignment between (Ta_2_O_5_)_*x*_(TiO_2_)_*1*−*x*_ and Si substrate was designed to enhance the memory performance by controlling the composition and dielectric constant of the charge-trapping layer and reducing the difference of the potentials at the bottom of the conduction band between (Ta_2_O_5_)_*x*_(TiO_2_)_*1*−*x*_ and Si substrate. The memory device with a composite charge storage layer (Ta_2_O_5_)_*0.5*_(TiO_2_)_*0.5*_ shows a density of trapped charges 3.84 × 10^13^/cm^2^ at ± 12 V, a programming/erasing speed of 1 µs at ± 10 V, a 8% degradation of the memory window at ± 10 V after 10^4^ programming/erasing cycles and a 32% losing of trapped charges after ten years. The difference among the activation energies of the trapped electrons in (Ta_2_O_5_)_*x*_(TiO_2_)_*1*−*x*_ CTM devices indicates that the retention characteristics are dominated by the difference of energy level for the trap sites in each TTO CTM device.

## Introduction

Charge trapping memory (CTM) devices like silicon-oxide-nitride-oxide -silicon (SONOS) type memory devices have attracted much attention in recent years. As one type of nonvolatile flash memories, charge trapping memory devices (CTM) using traditional storage dielectric materials (Si_3_N_4_) show excellent performance with high storage ability and are compatible with CMOS technology^1–4^, which means promising application in consumer electronics. With continuous down-scaling the cell dimension to obtain high data-storage density, high program/erase speeds, low operating voltage and low power consumption, some intrinsic limitations make this kind of memory rapidly approach the scaling limit, although 3D-architecture partly retards these challenges^5^. Various high-k dielectrics, such as HfO_2_, TiO_2_, ZrO_2_, Y_2_O_3_ and La_2_O_3_^6–9^, etc., as well as multilayer charge-trapping layer HfO_2_/Al_2_O_3_/HfO_2_ and ZrO_2_/Al_2_O_3_/ZrO_2_, have been employed to replace Si_3_N_4_ in SONOS devices to achieve a longer endurance and better retention property^10–16^. As a high-k dielectric, Al_2_O_3_ was also chosen as the tunneling and blocking layers in many similar memory devices due to its good chemical and thermal stability and large band offsets with Si^17, 18^.

Recently, high-k composite dielectrics have been employed as the charge-trapping layer, and its excellent charge-trapping efficiency was attributed to the high density of defect states formed due to the inter-diffusion between two kinds of high-k oxides^18–20^. It was also believed that by reducing the PBCB (potentials at the bottom of conduction band) between p-Si and high-k composite and increasing the dielectric constant of the high-k composite dielectric, the charge-trapping ability, programing/erasing speeds, and retention ability of the memory devices could be enhanced effectively.

TiO_2_ and Ta_2_O_5_ have been widely studied for high-k applications owing to their high permittivity, depending on the crystal structure and the method of deposition^21–23^. According to the calculation by J. Robertson by using the first principle theoretical method, the band gap of Ta_2_O_5_ is about 4.4 eV, and the PBCB between p-Si and Ta_2_O_5_ is about 0.3 eV^24^. TiO_2_(rutile) has a band gap of 3.1 eV, and the bottom of its conduction band is near the bottom of conduction band of Si, similar with that of BaTiO_3_^24^. So, the good performance of the CTM devices with high-k composite TiO_2_-Ta_2_O_5_ as the charge-trapping dielectric should be expected.

In this paper, we fabricated four CTM devices with the structure of Pt/Al_2_O_3_/(Ta_2_O_5_)_*x*_(TiO_2_)_*1*−*x*_/Al_2_O_3_/p-Si, and their memory properties were characterized.

## Experimental

**Figure 1:**
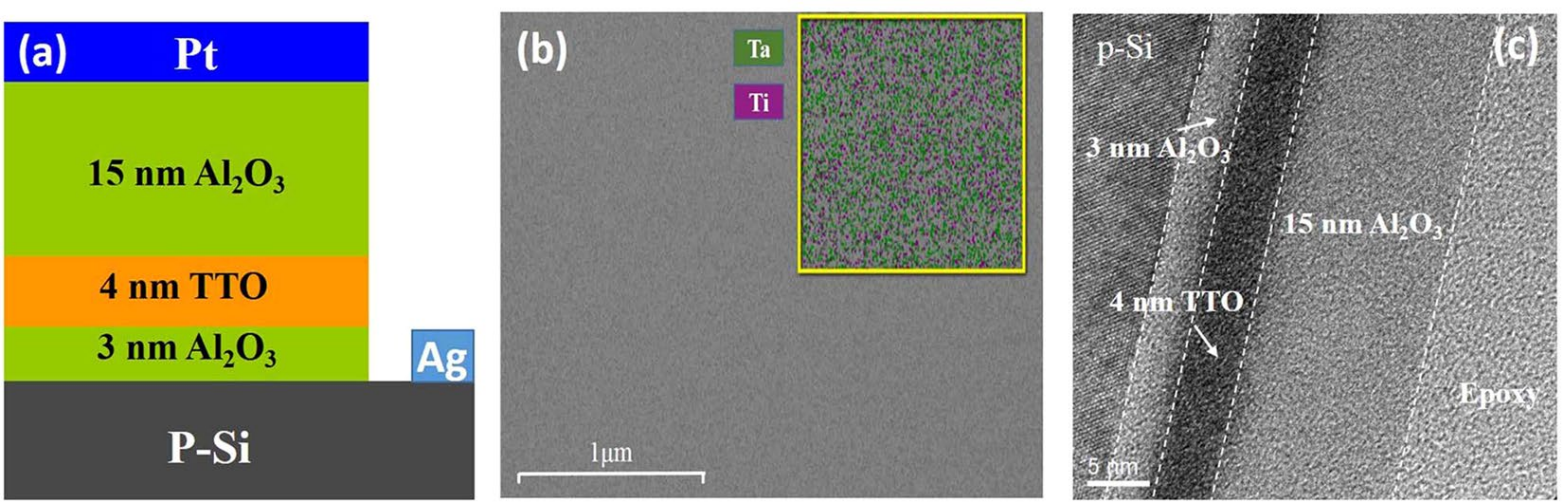
The schematic drawing of the structure of TTO CTM device (**a**), the surface morphology and element distribution of TTO(1:1) film (**b**), and the HRTEM cross-sectional image of TTO(1:1) CTM device (**c**).

The structure of (Ta_2_O_5_)_*x*_(TiO_2_)_*1*−*x*_ CTM devices was schematically drawn in Fig. 1a. Before fabricating the CTM devices, p-type (100) silicon wafers with a resistivity of 1–10 Ω cm were chosen as the substrates. The wafers were cleaned ultrasonically in alcohol and deionized water for 10 min respectively. Then the wafers were immersed in HF solution (HF: H_2_O = 1:10) for 30 s in order to remove surface oxide layers. After that, the wafers were rinsed by deionized water and dried by N_2_ for devices fabrication. A 3-nm Al_2_O_3_ thin film was deposited on wafers as the tunneling layer by using atomic layer deposition (ALD) system by using the precursors of trimethylaluminum (Al(CH_3_)_3_, TMA) and water reacting on the surface of wafers at 200 °C.

(Ta_2_O_5_)_*x*_(TiO_2_)_*1*−*x*_ sputtering ceramic targets were prepared by using Ta_2_O_5_ and TiO_2_ powders with x = 0.9, 0.75, 0.5, 0.25, and were named as TTO(9:1), TTO(3:1), TTO(1:1) and TTO(1:3), respectively. The raw powders were heated at 1300 °C for 8 h after well mixed by ball-milling. Then the mixed powder was ball-milled again and sintered in a box resistor stove at 1600 °C for 16 h. Eventually the obtained powder was extruded into a wafer shape with a 10-cm diameter. The TTO charge-trapping films were deposited by using RF-magnetron sputtering with a thickness of 4 nm at 100 W. The pressure of the deposition chamber was maintained at 2 Pa in a mixed atmosphere of argon and oxygen (flow ratio of 3:1). Then a 15-nm Al_2_O_3_ film was also deposited by using ALD technique as a blocking layer.

Pt top electrodes with a thickness of 100 nm and a diameter of 150 um were deposited on the fabricated samples with the aid of masks after rapid annealing at 200 °C for 60 s in N_2_. Ag adhesive was painted on the corner of the substrates as the bottom electrode.

To investigate the microstructure of four TTO films, TTO(9:1), TTO(3:1), TTO(1:1) and TTO(1:3) films with a thickness of 50 nm were fabricated on Si(001) substrates at 200 °C by using RF-sputtering technique. The microstructures of four TTO films were investigated by using XRD (Bruker D8 DISCOVER), and the surface morphology as well as the element composition in each TTO film were investigated by using scanning electron microscopy (SEM ZEISS ULTRA 55) and X-ray dispersive spectroscopy (EDS) techniques.

The microstructures of TTO CTM devices were observed by using high resolution transmission electron microscopy (HRTEM). The band alignments between TTO films and p-Si were calculated by analyzing the valence band spectra and O 1 s energy loss spectra obtained from X-ray photoelectron spectroscopy (XPS)^19^. The dielectric constants of TTO films and the memory characteristics of the fabricated devices were investigated by using Keithely 4200 semiconductor characterization system (Keithely 4200-SCS) at Cascade Summit 12000B-M platform.

## Results and Discussion

Please see the following production note; XRD patterns of four TTO films all show an amorphous structure. The surface morphology and the selected area element distribution of TTO(1:1) film were shown in Fig. 1b. It can be observed that TTO(1:1) film show a flat surface, and all the metallic elements (Ta and Ti) distribute in the film uniformly. Due to the high concentration of O in TTO(1:1) film which could affect the mapping of other elements such as Ta and Ti, O mapping in TTO(1:1) films were omitted. Similar flat surface morphology and the uniform element distribution were also observed in other TTO films. The cross-sectional morphologies of four TTO CTM devices were observed by using HRTEM. It was observed that all devices show similar morphology. Figure 1c shows the cross-sectional morphology of TTO(1:1) CTM device. The interface between p-Si substrate and the tunneling layer Al_2_O_3_ is quite sharp. The thicknesses of the tunneling layer, the charge trapping layer and the blocking layer are about 3 nm, 4 nm and 15 nm, respectively. Compared with Si substrate, Al_2_O_3_ films both in the tunneling layer and the blocking layer as well as the TTO film in the charge trapping layer show an amorphous structure, favorable to the performance of CTM devices.

To measure the dielectric constant of TTO films, the capacitance structure of Pt/TTO(9:1)/Pt, Pt/TTO(3:1)/Pt, Pt/TTO(1:1)/Pt and Pt/TTO(1:3)/Pt by using rf- and dc-sputtering techniques, respectively, in which the thickness of the TTO dielectric film is about 30 nm. The dielectric constants of TTO(9:1), TTO(3:1) and TTO(1:1) films were calculated as about 19, 30, 44 and 62, respectively, indicating that the dielectric constant of TTO film increases with the increase of TiO_2_ composition.

**Figure 2:**
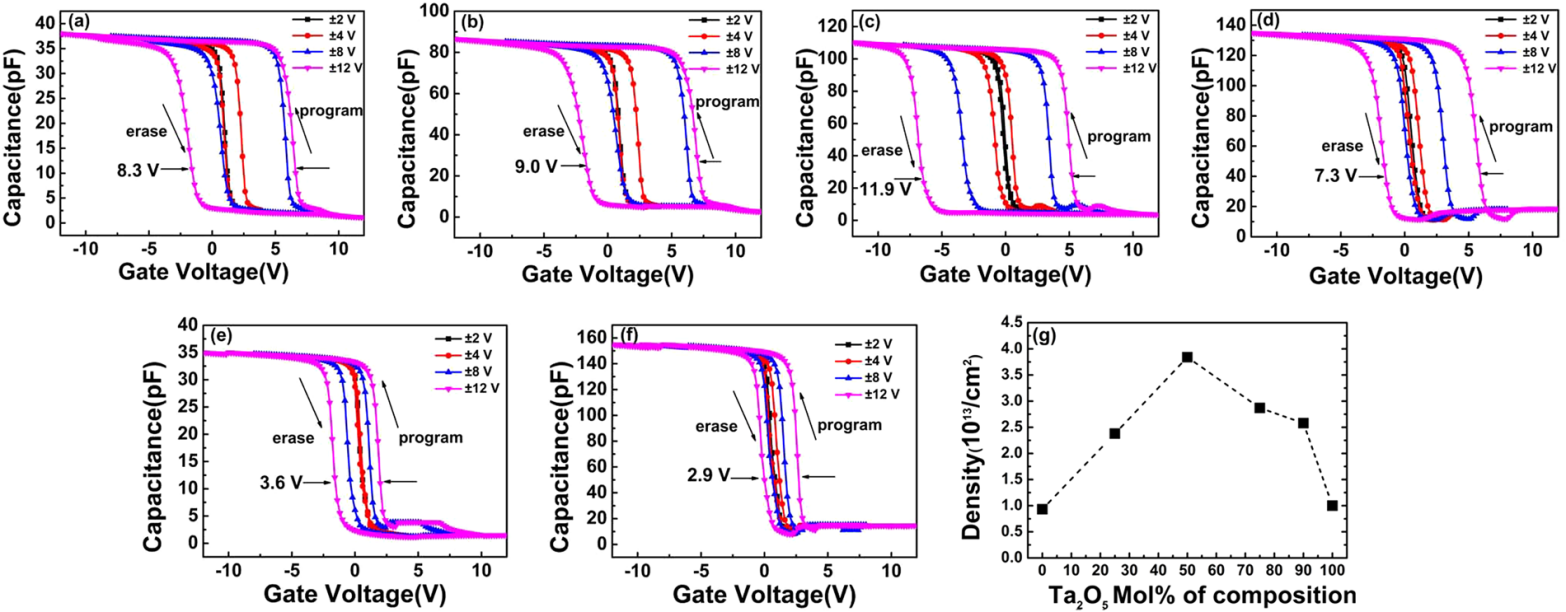
The capacitance-voltage (C-V) curves for TTO(9:1) (**a**), TTO(3:1) (**b**), TTO(1:1) (**c**), TTO(1:3) (**d**), Ta_2_O_5_ (**e)**, TiO_2_ (**f**) CTM devices, and the densities of trapped charges under the sweeping voltage of ± 12 V (**g**).

Figure 2a,b,c and d show the applied gate-voltage dependence of the capacitances for four TTO CTM devices. With the increase of sweeping gate voltage at a frequency of 1 MHz, the memory windows increase quickly. In a sweeping cycle of gate voltage from −12 V to +12 V and then back to −12 V, the memory windows (Δ*V*_*FB*_) reach to 8.3 V, 9.0 V, 11.9 V and 7.3 V for TTO(9:1), TTO(3:1), TTO(1:1) and TTO(1:3) CTM devices, respectively. The density of trapped charges in a CTM device can be estimated by using the formula^25, 26^:

1 $${\rm{\Delta }}{V}_{FB}=-\frac{\,q{N}_{t}}{C}$$

Where *C* is capacitance per unit area of the dielectric from charge traps to Pt gate, Δ*V*_*FB*_ is the memory window, *q* is the electron charge. Here, the values of *C* for TTO(9:1), TTO(3:1), TTO(1:1) and TTO(1:3) CTM devices are calculated as 88.3 pF, 90.3 pF, 91.4 pF, and 92.1 pF, respectively, and the difference among them should be attributed to the different dielectric constants due to the different composition of Ta_2_O_5_ and TiO_2_. With the following parameters: d_2_ = 15 nm, d_t_ = 4 nm and *ε*_*Al*2*O*3_ = 9, the densities of trapped charges in four TTO CTM devices corresponding to Fig. 2a,b,c and d were estimated as about 2.58 × 10^13^/cm^2^, 2.87 × 10^13^/cm^2^, 3.84 × 10^13^/cm^2^ and 2.38 × 10^13^/cm^2^, respectively, as shown in Fig. 2g.

To make a comparison, the CTM memory structures Pt/Al_2_O_3_/Ta_2_O_5_/Al_2_O_3_/p-Si and Pt/Al_2_O_3_/TiO_2_/Al_2_O_3_/p-Si with the same structural parameters with those of TTO CTM devices were also fabricated. As shown in Fig. 2e, a memory window of about 3.6 V was obtained in a sweeping cycle of gate voltage from −12 V to +12 V for Ta_2_O_5_ CTM device, corresponding to a density of trapped charges 1.0 × 10^13^/cm^2^, much lower than that obtained in TTO CTM devices. From Fig. 2f, a density of trapped charges 9.31 × 10^12^/cm^2^ was obtained in TiO_2_ CTM device, which is also much lower than that in TTO(1:1) CTM devices.

A capacitance structure Pt/Al_2_O_3_/Si(100), in which the thickness of Al_2_O_3_ is the same as the total thickness of the tunneling layer Al_2_O_3_, the charge-trapping layer TTO and the blocking layer Al_2_O_3_, was also fabricated to investigate the charge-trapping effect at the interface Al_2_O_3_/Si and inside Al_2_O_3_ tunneling and blocking layers. A small memory window of about 1.0 V was obtained in a sweeping cycle of gate voltage from −12 V to +12 V. The density of trapped charges for the capacitance structure Pt/Al_2_O_3_/Si(100) can be estimated by using the formula:

2 $${N}_{t}=\frac{{C}_{acc}\cdot {\rm{\Delta }}{V}_{FB}}{q\cdot A}$$

Where *C*_*acc*_ is the accumulative capacitance of the structure, Δ*V*_*FB*_ is the memory window, *q* is the electron charge and *A* is the area of Pt electrodes. Here *A* is 1.77 × 10^−4^ cm^2^, correspondingly the density of trapped charges is about 1.12 × 10^12^/cm^2^, much lower than that obtained in TTO CTM devices. The observed electronic states should be ascribed to the inter-diffusion at the interface Si/Al_2_O_3_. So it can be concluded that in TTO CTM devices the charges are mainly trapped in TTO layer.

In TTO(1:1) film with the most effective mixing between Ta_2_O_5_ and TiO_2_, the largest density of defect states should be expected, thus TTO(1:1) CTM device gets the largest density of trapped charges, similar with that observed in (Ta_2_O_5_)_*x*_(Al_2_O_3_)_*1*−*x*_ system^19^. In contrast, there exists the least mixing between Ta_2_O_5_ and TiO_2_ in TTO(9:1) film, so the lowest density of trapped charges should be expected in TTO(9:1) CTM devices. Although the higher dielectric constant due to the increase of TiO_2_ content favors the density of trapped charges in TTO(1:3) CTM device^20^, a lower density of trapped charges was obtained as compared with that in TTO(1:1) CTM device. It should be attributed to the less effective mixing between Ta_2_O_5_ and TiO_2_ in TTO(1:3) charge-trapping layer. In the following part, only memory properties for TTO(9:1), TTO(3:1) and TTO(1:1) CTM devices will be discussed.

Although the definite clarification on the origination of the electronic states formed at the interface Ta_2_O_5_/TiO_2_ is difficult, the studies on the two-dimensional electron gases at oxide interface of epitaxial perovskite hetero-structure can give us some clues^27, 28^. It was believed that electrostatic boundary conditions become a dominant factor controlling the atomic and electronic structure at solid-solid interface. The electron re-distribution help the interface realize the electrostatic equivalence, resulting in a high density of electronic states at the interface. Similar two-dimensional electron gas was also realized at amorphous oxides/SrTiO_3_ hetero-structural interface, such as at amorphous LaAlO_3_/SrTiO_3_(001), amorphous YAlO_3_/SrTiO_3_(001) and amorphous Al_2_O_3_/SrTiO_3_(001) interfaces^29^. Correspondingly, in case of the interface Ta_2_O_5_/TiO_2_ the high density of the defect states should be ascribed to the electron re-distribution between the cations with different valence and anion (oxygen) due to the appearance of the dangling bonds formed at the surface of each high-k oxide.

**Figure 3:**
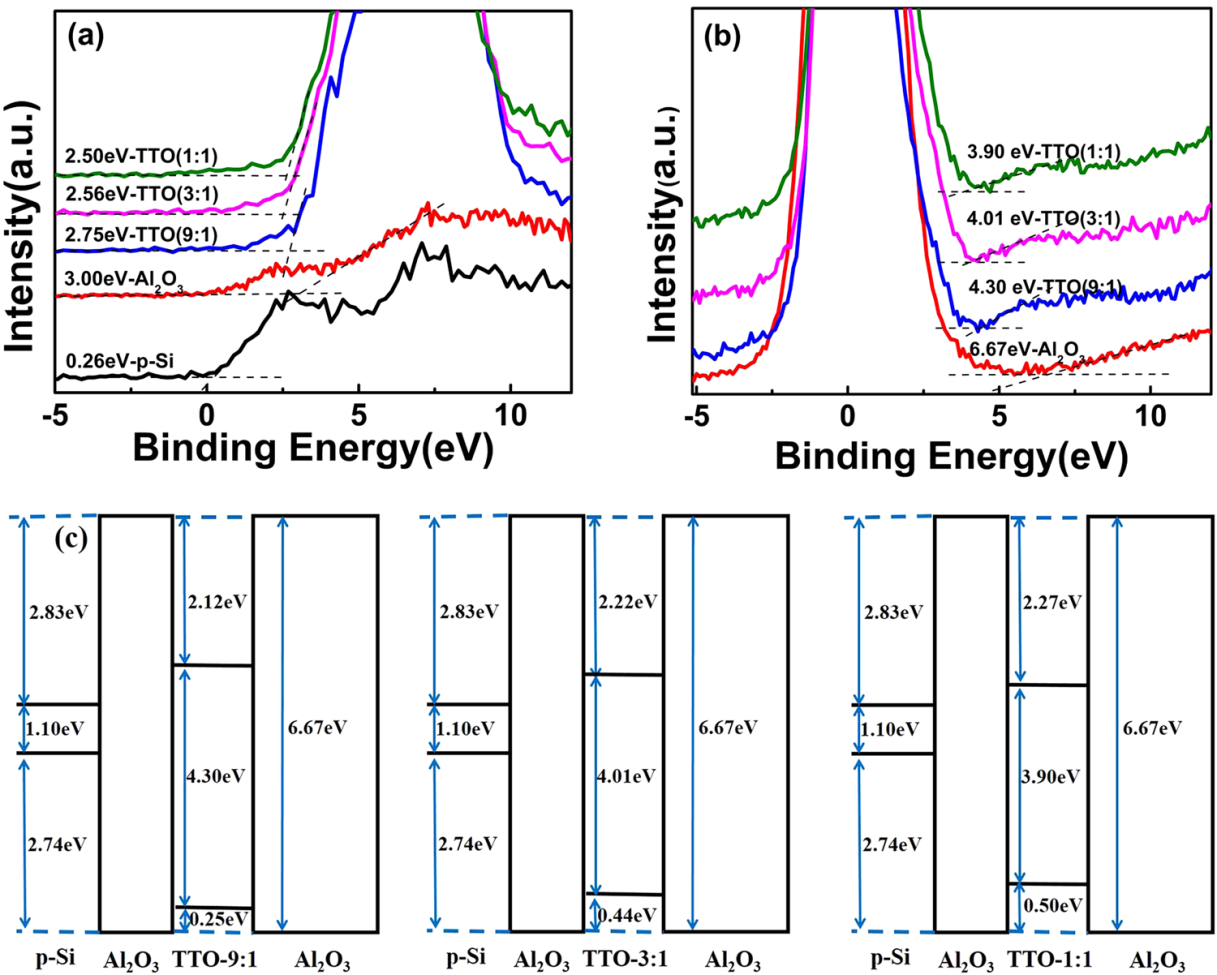
The valence band spectra of TTO composite samples, Al_2_O_3_ and p-Si substrate (**a**), O 1 s energy loss spectra of TTO composite samples and Al_2_O_3_ (**b**), and the band alignments of TTO CTM devices (**c**).

To investigate the Ta_2_O_5_-TiO_2_ composition dependence of the band alignments between p-Si and TTO films, the valence band spectra and O 1 s energy loss spectra for TTO films were measured by using XPS. Figure 3a shows the valence band spectra of TTO(9:1), TTO(3:1) and TTO(1:1) films as well as Al_2_O_3_ films and Si substrate, respectively. The valence band maximum (VBM) of each film can be roughly estimated by linear extrapolating from the edge of valence band (VB) to the background level^30^. As shown in Fig. 3a, the value of VBM for Si (${E}_{VBM}^{Si}$) is 0.26 eV, and those for Al_2_O_3_ (${E}_{VBM}^{A{l}_{2}{O}_{3}}$), TTO(9:1) (${E}_{VBM}^{{\rm{TTO}}(9:1)}$), TTO(3:1) (${E}_{VBM}^{{\rm{TTO}}(3:1)}$), TTO(1:1) (${E}_{VBM}^{{\rm{TTO}}(1:1)}$) are 3.0 eV, 2.75 eV, 2.56 eV, and 2.5 eV, respectively. The valence band offset (VBO) of Al_2_O_3_/Si (${{\rm{\Delta }}E}_{v}^{A{l}_{2}{O}_{3}/Si}$), TTO(9:1)/Al_2_O_3_, TTO(3:1)/Al_2_O_3_ and TTO(1:1)/Al_2_O_3_ are calculated as 2.74 eV, 0.25 eV, 0.44 eV, and 0.5 eV, respectively, by using the following formula:

3 $${{\rm{\Delta }}{\rm{E}}}_{v}^{A{l}_{2}{O}_{3}/Si}={E}_{VBM}^{A{l}_{2}{O}_{3}}-{E}_{VBM}^{Si}$$

4 $${\rm{\Delta }}{E}_{v}^{A{l}_{2}{O}_{3}/TTO}={E}_{VBM}^{A{l}_{2}{O}_{3}}-{E}_{VBM}^{TTO}$$

The band gaps of each high-k dielectric could be obtained by using a linear fitting method by analyzing the onset of a loss spectrum from the O 1 s energy loss signal^30^. The O 1 s energy loss spectra of Al_2_O_3_, TTO(9:1), TTO(3:1) and TTO(1:1) were shown in Fig. 3b, respectively, and their band gaps were determined as 6.67 eV, 4.30 eV, 4.01 eV, and 3.90 eV respectively. The band alignments of three TTO CTM devices were shown schematically in Fig. 3c, which is helpful for us to understand their memory performance.

**Figure 4:**
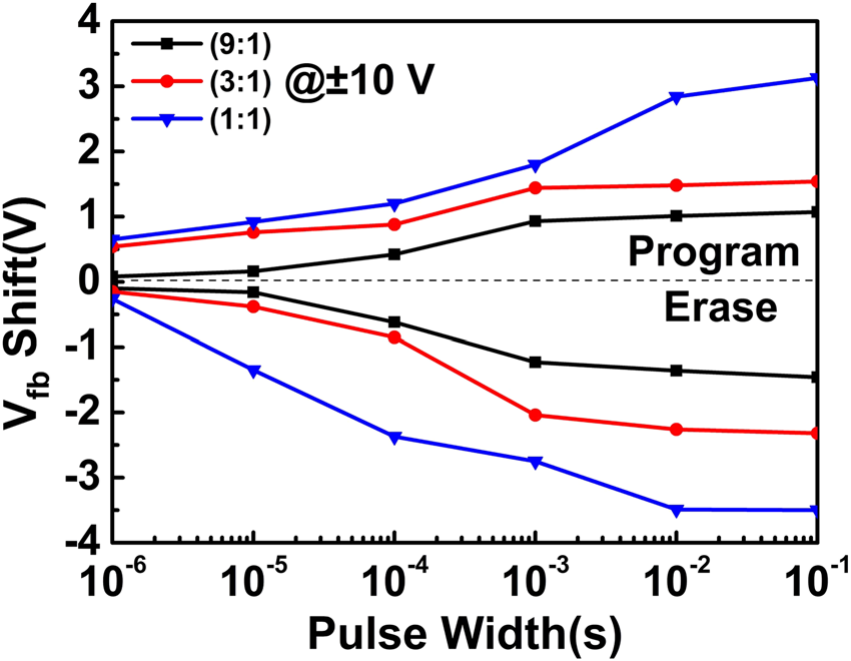
The programing/erasing (P/E) characteristics of TTO(9:1), TTO(3:1) and TTO(1:1) CTM devices.

Figure 4 shows the Program/Erase (P/E) characteristics of TTO(9:1), TTO(3:1) and TTO(1:1) CTM devices. In these experiments, a series of voltage pulses with an amplitude of ± 10 V and different pulse widths from 10^−6^ s to 0.1 s were applied to TTO CTM devices. As a response to the applied voltage pulse, there should be a flat-band-voltage shift (Δ*V*_*FB*_) in the C-V curve of TTO CTM device, representing the amount of electrons programed into or erased from the charge trapping layer. There exists obvious Δ*V*_*FB*_ in the C-V curves of all devices at an applied voltage with a pulse width of 10^−6^ s, and TTO(1:1) CTM device gets the largest Δ*V*_*FB*_ of 0.91 V, much larger than that obtained in TiO_2_-Al_2_O_3_ CTM device as well as Si_3_N_4_, Ta_2_O_5_, HfO_2_ and ZrO_2_ CTM devices^18, 31–33^. With the increase of the pulse width of the applied voltage, the values of Δ*V*_*FB*_ increase quickly in all devices, and Δ*V*_*FB*_ in TTO(1:1) CTM device at the applied voltage with a pulse width of 0.1 s is about 7.49 V.

The differences among the P/E speeds of three TTO CTM devices should be attributed to their special band alignments as shown in Fig. 3c and individual density of trapped charges. The heights of the potential barriers between p-Si and the charge-trapping dielectric for three TTO CTM devices can be roughly determined by comparing the bottoms of conduction band (or Fermi level) between p-Si and TTO dielectrics. From Fig. 3c, there clearly exists the lowest height of the barrier between TTO(1:1) and p-Si. At a large enough positive voltage applied between the top and bottom electrodes, part of the voltage is employed for electrons to overcome the barrier between p-Si and TTO composite dielectric. The left part of the applied voltage provides electrons with dynamic energy to tunnel from p-Si through tunneling layer Al_2_O_3_ to TTO charge-trapping layer, and then trapped in defect states. The electrons with a larger dynamic energy should have a larger probability to tunnel through the tunneling layer Al_2_O_3_. It was calculated that the injection current density of electrons in the programming process for a floating-gate memory device is proportional to the trapping density and the function $\exp (-\frac{{\rm{q}}{\varnothing }_{1}}{{\rm{KT}}})$, where *Φ*_*1*_ is the barrier height^25, 26^. In TTO CTM devices the similar physical process should be expected. TTO(1:1) composite with the largest trapping density and the lowest barrier height among three TTO composites enables TTO(1:1) CTM device to get the largest injection current density at the same applied gate voltage, resulting in the largest ΔV_FB_ as shown in Fig. 4. In contrast, TTO(9:1) CTM device gets the lowest ΔV_FB_, in which TTO(9:1) has the lowest trapping density and the largest barrier height. In the erasing process (discharging), TTO(1:1) CTM device will also get the largest inverse current density due to the lowest barrier height and the largest amount of trapped charges in TTO(1:1) dielectric, thus resulting in the largest ΔV_FB_.

**Figure 5:**
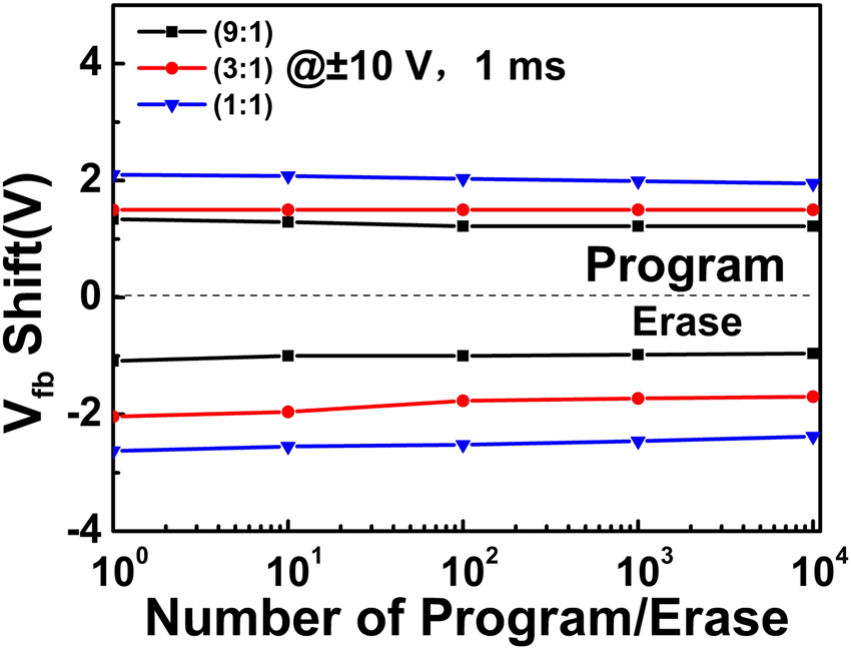
The endurance properties of TTO(9:1), TTO(3:1) and TTO(1:1) CTM devices.

**Figure 6:**
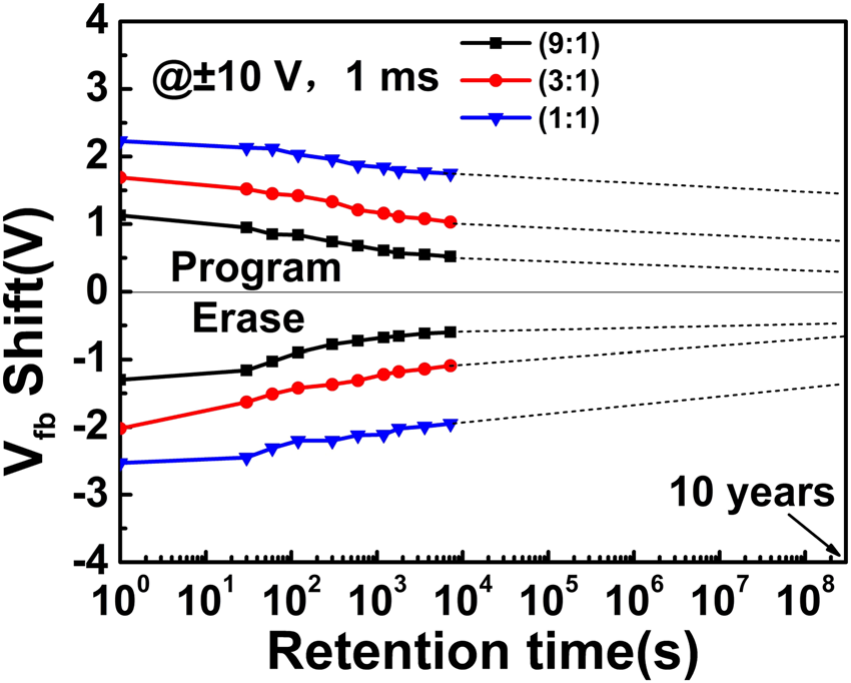
The retention properties of TTO(9:1), TTO(3:1) and TTO(1:1) CTM devices at room temperature.

Figure 5 shows the endurance properties of three TTO CTM devices. All devices show excellent endurance characteristics, and after a P/E operation cycles of 1 × 10^4^ the degradations are all less than 10%. The retention properties of TTO(9:1), TTO(3:1) and TTO(1:1) CTM devices were investigated under a sweeping gate voltage with an amplitude of ± 10 V and a pulse width of 1 ms, as shown in Fig. 6. In order to get the tendency of data retention after ten years, the curves of three TTO CTM devices were extended to 3 × 10^8^ s. The TTO(1:1) CTM device with the largest density of trapped charges shows the best retention property. Only 32% of its trapped charges were lost after ten year in The TTO(1:1) CTM device at room temperature, while about 70% of the trapped charges were lost in the TTO(9:1) CTM device. Although it was believed that the deep trap level in Ta_2_O_5_ favors the retention property^34^, the retention properties of the TTO CTM devices become better with the decrease of Ta_2_O_5_ composition as shown in Fig. 6. In addition, a larger density of trapped charges in the TTO charge-trapping layer should lead to a larger inverse electric field between Si-substrate and the TTO charge-trapping layer, resulting in a larger probability of tunneling back to Si-substrate from the TTO charge-trapping layer for the trapped electrons. The anomalies among the retention properties of three TTO CTM devices also should be ascribed to the special band alignment of each device.

Different with the programming and erasing processes in which the electron tunneling is driven by the applied electric field, the charge loss in the retention property in TTO CTM devices as shown in Fig. 6 is driven by thermal excitation. The trapped electrons in TTO charge-trapping layer of TTO(1:1) CTM device with a lower bottom of the conduction band have a lower potential than those in other two TTO CTM devices. The energy needed to thermally excite electron back to Si substrate in TTO(1:1) CTM device is larger than those in other two TTO CTM devices, leading to few electrons lost. The retention properties of TTO CTM devices should be dominated by the potential at the bottom of the conduction band.

**Figure 7:**
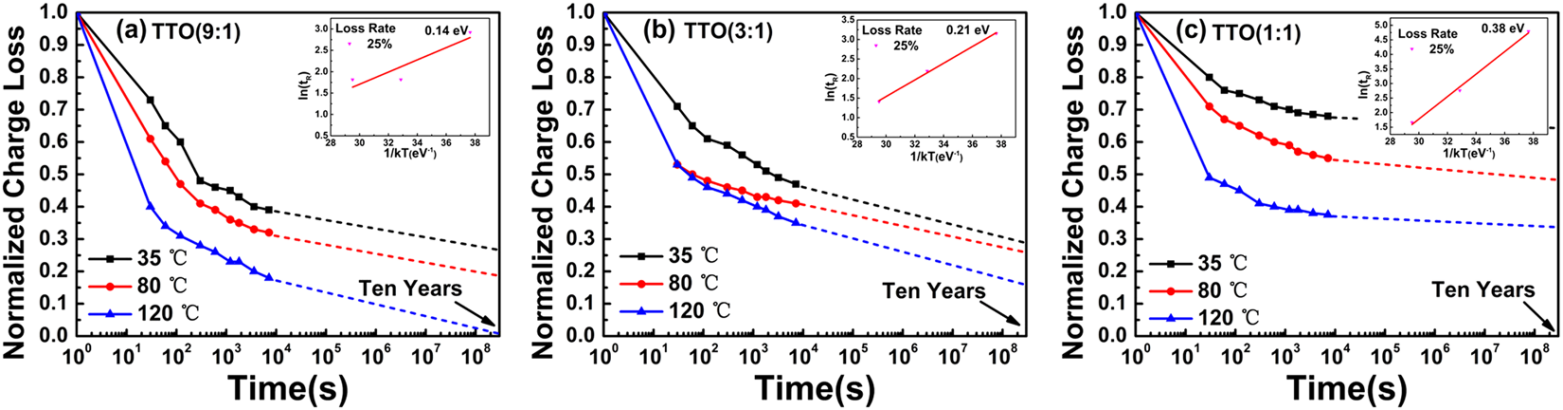
Normalized charge loss characteristics of the (**a**) TTO(9:1), (**b**) TTO(3:1) and (**c**) TTO(1:1) CTM devices at 35, 80, 120 °C. The insets are Arrhenius plots of retention time and reciprocal temperature.

To further investigate the charge loss mechanism in TTO CTM devices, the retention characteristics of TTO(9:1), TTO(3:1) and TTO(1:1) CTM devices at different temperatures have been measured to calculate the activation energy, as shown in Fig. 7a,b and c, respectively. Here the retention time model with linear variations according to the temperature, which employs a T extrapolation model by Salvo *et al*., was considered^35, 36^. The inset figures in Fig. 7a,b and c show the Arrhenius plots for the retention time characteristics of charge loss ratio for TTO(9:1), TTO(3:1) and TTO(1:1) CTM devices, respectively. Based on the temperature dependence of the charge loss, the activation energies of electrons trapped in TTO(9:1), TTO(3:1) and TTO(1:1) layers were estimated as about 0.14 eV, 0.21 eV and 0.38 eV, respectively, by using the formula:

5 $${E}_{a}=\partial \,\mathrm{ln}({t}_{R})/\partial (\frac{1}{kT})$$

Where *E*_*a*_, *t*_*R*_, *K*, and *T* are the activation energy, the retention time (which is needed for the amount of the trapped charges to degrade to 25% the initial value), Boltzman constant and the temperature, respectively. It means that the charges are deeply trapped at the interface states in all TTO CTM devices. The difference among the activation energies of the trapped electrons in three TTO CTM devices should be ascribed to the difference of energy level for the trap sites in each TTO CTM device, which leads to the difference of the retention characteristics of three TTO CTM devices.

## Conclusion

In summary, the CTM devices with Ta_2_O_5_-TiO_2_ composite as the charge-trapping layer have been fabricated by using sputtering and ALD techniques. By designing a proper mixing ratio between Ta_2_O_5_ and TiO_2_ and a low difference of the potentials at the bottom of the conduction band between the charge-trapping layer and Si substrate, a stable microstructure, a large density of trapped charges, a fast P/E speed and good endurance and retention properties were obtained in TTO(1:1) CTM device. The distinguished memory performance was dominated by its high density of defect states in TTO dielectric and the special band alignment between TTO dielectric and Si substrate. With a simple structure, a prominent charge-trapping capability and good reliability of data storage, the TTO(1:1) CTM device should be one of the possible selections for non-volatile memory applications in the future.
